# Prompt engineering for digital mental health: a short review

**DOI:** 10.3389/fdgth.2024.1410947

**Published:** 2024-06-12

**Authors:** Y. H. P. P. Priyadarshana, Ashala Senanayake, Zilu Liang, Ian Piumarta

**Affiliations:** Ubiquitous and Personal Computing Lab, Faculty of Engineering, Kyoto University of Advanced Science (KUAS), Kyoto, Japan

**Keywords:** prompt engineering, digital mental health, natural language processing, large language models, generative artificial intelligence

## Abstract

Prompt engineering, the process of arranging input or prompts given to a large language model to guide it in producing desired outputs, is an emerging field of research that shapes how these models understand tasks, process information, and generate responses in a wide range of natural language processing (NLP) applications. Digital mental health, on the other hand, is becoming increasingly important for several reasons including early detection and intervention, and to mitigate limited availability of highly skilled medical staff for clinical diagnosis. This short review outlines the latest advances in prompt engineering in the field of NLP for digital mental health. To our knowledge, this review is the first attempt to discuss the latest prompt engineering types, methods, and tasks that are used in digital mental health applications. We discuss three types of digital mental health tasks: classification, generation, and question answering. To conclude, we discuss the challenges, limitations, ethical considerations, and future directions in prompt engineering for digital mental health. We believe that this short review contributes a useful point of departure for future research in prompt engineering for digital mental health.

## 1 Introduction

**Figure 1:**
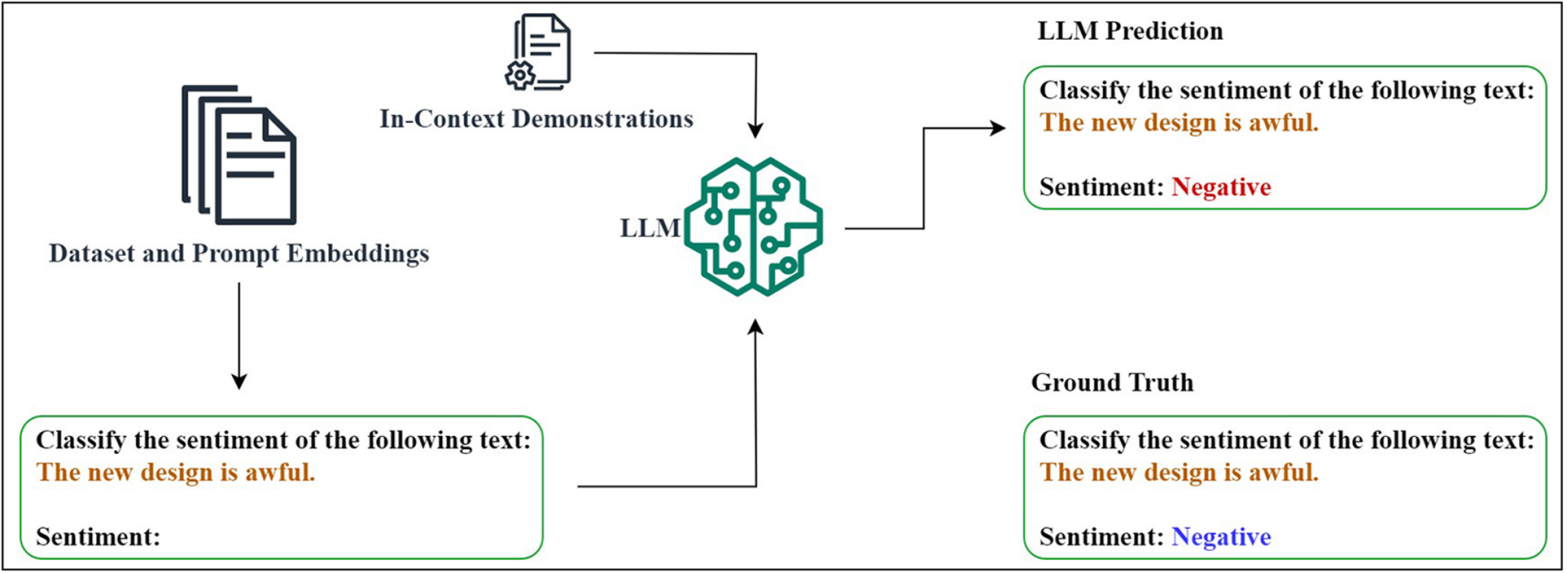
Prompt-based LLM fine-tuning for sentiment analysis.

Even though adapting general-purpose pre-trained large language models (LLMs) to various natural language processing (NLP) tasks such as sentiment analysis has gained a significant attention due to its task-specific fine-tuning capabilities (1), this approach still demands high computational resources and task-specific labelled corpora which make it inappropriate for improving few-shot task performance in complex systems (2). Prompt engineering (PE) has therefore become state-of-the-art (SOTA) for casting various NLP-driven downstream tasks into a general-purpose LLM format (3). As shown in Figure 1, parameter-efficient prompt engineering methods have gained superiority by prepending prompt embeddings to input data while keeping the majority of the LLM frozen (4).

On the other hand, better early identification of human mental disorders has become a vital necessity due to the significant skilled labor requirement for clinical diagnosis-based approaches (5). Even though a few LLM-driven approaches have been introduced for mental disorder detection, fine-tuning their performance is hampered by the limited scalability of the models (6). PE-based methods have recently shown significant improvement for the detection of mental disorders such as depression and anxiety using user-generated text (7).

In this short review, we focused on recently published articles since 2020 by querying four online databases (ACM Digital Library, PubMed, Google Scholar, and IEEE Xplore), using keywords such as “Prompt Engineering,” “Deep Learning for Mental Health,” “Deep Learning for Digital Mental Health,” “In-context Learning,” “Prompt Tuning,” “Instruction Prompt Tuning,” “In-domain Prompting,” “Out-of-domain Prompting,” “Out-of-distribution Prompting” “Chain-Of-Thought Prompting,” “N-shot Prompting,” “Large Language Models,” “Mental Health Classification,” and “Mental Health Reasoning,” related to methods, types, and applications on PE for digital mental health (DMH). The articles were compiled in a spreadsheet and then were filtered based on DMH type, PE type, PE method, PE task, LLMs used, and input data. To our knowledge, this is the first such review of PE-based methods for DMH. We summarize the overall review in Table 1 and discuss types of PE in Section 2. PE-based methods for DMH and applications are presented in Sections 3 and 4, respectively. Limitations, challenges, and future directions are described in Section 5.

**Table 1:** Summary of the papers selected in this short review, classified into DHM type (D, depression; Anx, anxiety; ST, suicidal thoughts; CD, cognitive distortion; S, stress) PE types, PE task, data, and methods.

Papers	DMH type	PE type	PE method	PE task	LLMs	Data	Results
Czejdo et al. (8)	D, Anx	N-shot COT	ICL	In-domain	GPT-3 Davinci	Q&A Summarization	Davinci’s capability in n-shot Q&A
Tlachac et al. (9)	D, Anx	N-shot	ICL	In-domain	GPT-3	Scripted audio (SA) Unscripted audio (USA)	D F1 (SA)—0.746 D F1 (USA)—0.691 Anx F1 (SA)—0.667 Anx F1 (USA)—0.63
Ji (10)	ST	N-shot	IPT	In-domain	BERT MBERT	Reddit posts Weibo posts	F1 (BERT)—0.571 F1 (MBERT)—0.61
Qi et al. (11)	ST, CD	N-shot	ICL PT	In-domain	GLM GPT-3.5 GPT-4	Weibo posts Zoufan blogs	ST F1 (GLM)—0.722 ST F1 (GPT-4)—0.75 CD F1 (GLM)—0.17 CD F1 (GPT-4)—0.32
Yang et al. (12)	D, S, ST	N-shot COT	ICL PT	In-domain	ChatGPT GPT-3 LLaMA	Reddit posts CLPsych15 Dreaddit T-SID	D F1 (GPT-3)—0.831 S F1(ChatGPT)—0.85 ST F1 (LLaMA)—0.54
Amin et al. (13)	D, ST	N-shot	ICL	In-domain	ChatGPT	Reddit posts Sentiment-140	D Accuracy—0.855 ST Recall—0.912
Lamichhane (14)	D, S, ST	Few-shot	ICL	In-domain	ChatGPT	Reddit posts Dreaddit	D F1—0.73 S F1—0.86 ST F1—0.37
Xu et al. (15)	D, S, ST	N-shot	ICL	In-domain	GPT-4 FLAN-T5 LLaMA	DepSeverity SDCNL CSSRS	D F1 (GPT-4)—0.719 S F1 (FLAN-T5)—0.67 ST F1 (LLaMA)—0.72
Guo et al. (16)	D	N-shot	ICL	In-domain	PTDD	DAIC-WOZ	Accuracy—0.69 F1—0.60
Ghanadian et al. (17)	ST	N-shot	ICL	In-domain	ChatGPT	Reddit UMD	Accuracy—0.88 F1—0.73
Yang et al. (18)	D	N-shot	ICL	In-domain	ChatGPT GPT-4 LLaMA MLLaMA	IMHI	F1 (ChatGPT)—0.71 F1 (GPT-4)—0.781 F1 (LLaMA)—0.615 F1 (MLLaMA)—0.83
Qin et al. (19)	D	N-shot COT	ICL	Out-of-distribution	BERT ChatGPT GPT-3	Weibo posts Twitter MDD	F1 (BERT)—0.587 F1 (ChatGPT)—0.79 F1 (GPT-3)—0.851
Ramos et al. (20)	D	N-shot	ICL	In-domain	BERT GPT-3.5	SetembroBR	F1 (BERT)—0.65 F1 (GPT-3.5)—0.66
Zhang et al. (21)	D	N-shot	ICL	In-domain	BERT T5 FGPL	DAIC-WOZ	F1 (BERT)—0.7407 F1 (T5)—0.75 F1 (FGPL)—0.7692
Malhotra et al. (22)	D, Anx	N-shot	ICL	Out-of- distribution	BERT MBERT	Twitter posts	F1 (BERT)—0.866 F1 (MBERT)—0.888
Agrawal (23)	D	COT	ICL	Out-of-distribution	GPT-4 LLaMA Gemini	DAIC-WOZ Reddit MHD	F1 (GPT-4)—0.74 F1 (LLaMA)—0.69 F1 (Gemini Pro)—0.66
Chiu et al. (24)	S	N-shot	ICL	In-domain	GPT-3 GPT-3.5 GPT-4	Therapy conversations HOPE	F1 (GPT-3)—0.496 F1 (GPT-3.5)—0.371 F1 (GPT-4)—0.577

## 2 Types of prompt engineering

### 2.1 N-shot prompting

N-shot prompting is an NLP technique for guiding LLMs to perform specific tasks with “*N*” examples in the prompt to understand the task. It enables in-context learning of LLMs for better performance with minimal additional training (2). Based on the in-context (“*N*”) examples provided to LLMs, n-shot prompting can be further separated into zero-shot prompting and few-shot prompting. Zero-shot prompting has shown some promising results in performing well-designed prompt-driven non-complex tasks such as information retrieval, language translations, and question answering, without corresponding task-specific examples where the model must rely on its pre-existing knowledge and the task description in the prompt (25). Recent studies such as (13) and Lamichhane (14) have shown the capability of zero-shot prompting in ChatGPT for depression and suicidal detection. Few-shot prompting, on the other hand, performs well in complex tasks such as custom text generation and domain-specific question answering, using in-context examples (typically between two and five) along with task-specific prompts to steer an LLM for better understanding the task and to produce more accurate and contextually appropriate responses, compared to zero-shot prompting (26). Mental-RoBERTa (27) and Mental-FLAN-T5 (15) have been used to classify depression, stress, and suicidal thoughts using few-shot prompting.

### 2.2 Chain-of-thought (COT) prompting

COT prompting is an NLP technique to improve the reasoning capabilities of LLMs using structured prompts and immediate reasoning steps. In contrast with the application of LLMs to classification tasks using N-shot prompting, COT prompting helps the LLM to breakdown complex problems into manageable tasks and improves its ability to handle tasks using multi-step problem solving and explanation generation (28). Assessing the accuracy of LLM-generated explanations for mental health is critical. Kojima et al. (29) modified the vanilla prompt design using COT prompting to enhance the reasoning capability of GPT-3.5 and GPT-4 in metal health contexts. Englhardt et al. (30) suggested a novel approach based on multi-model time-series data to improve the reasoning abilities of LLMs for detecting depression and anxiety. A few studies have shown the explainability of LLMs in the context of mental health using end-user applications such as chatbots (31). Wang et al. (32) proposed a new COT framework to assess the mental status of users following multiple COT prompting reasoning steps in both zero-shot and few-shot settings. Chen et al. (33) introduced an enhanced version of COT prompting called Diagnosis of Thought prompting, a conceptual approach similar to COT prompting but focused more on understanding and validating the thought process behind the LLM's responses, to detect cognitive distortions. Although COT prompting improves the LLM's ability to handle complex tasks compared to N-shot prompting, the quality of prompts can limit the effectiveness.

## 3 Methods of prompt engineering

### 3.1 In-context learning (ICL)

ICL is the simplest PE method to adapt the knowledge of GPT-3 to solve a new, semantically similar tasks without additional explicit training using in-context examples, also known as demonstrations, inspired by the knowledge transferability of the human brain to new tasks using few instructions (2). Liu et al. (34) showed the importance of dynamically retrieved demonstrations over random demonstrations for natural language generation (NLG) tasks. Hayati et al. (35) explored the few-shot capability of GPT-3 for depression detection using contextually similar demonstrations. Su et al. (36) further demonstrated the mental health reasoning capabilities of LLMs using a new ICL framework. Fu et al. (37) introduced a commonsense-based response generation method by enhancing the explainability of ChatGPT and T5 models in the context of mental health using domain-specific demonstrations. Recently (38), developed the *GoodTimes* app, a personalized conversational and storytelling tool for reminiscence therapy, using the ICL-based reasoning capabilities of SOTA NLP models. As shown in Table 1, ICL-based N-shot prompting shows significant results in depression, stress, and suicidal thought detection (14, 15). Even though multiple DMH studies have been conducted for contextually similar knowledge transfer using ICL-based techniques, adapting knowledge to contextually dissimilar tasks is yet to be achieved due to limitations such as the lack of relevant contextual cues, differences in dissimilar tasks structures, limited generalization of LLMs to transfer knowledge, and the complexity of creating effective prompts for contextually dissimilar tasks (39).

### 3.2 Prompt tuning (PT)

Considering the limitations of ICL, soft continuous prompts were proposed to enhance the in-context capability of GPT-3 to execute a new task by adapting a few parameters while keeping the majority of the LLM frozen (4). Blair et al. (40) introduced a few-shot PT-based domain transfer technique for named entity disambiguation in mental health news articles. Li et al. (41) suggested novel PT-based optimization methods and a reinforcement learning framework for GPT-4 which can be used for mental health related NLG tasks. Spathis et al. (42) used PT-based evaluation protocols such as zero-shot inference to work with temporal stress levels data. According to Table 1, PT-based N-shot prompting performs better than ICL-based N-shot and COT prompting in suicidal thoughts and cognitive distortion detection (11, 12). PT-based methods are still unstable for scaling LLMs even though such methods outperform ICL-based approaches due to optimization challenges, LLM complexity, and the absence of sufficient contextual information for LLM generalization (43).

### 3.3 Instruction prompt tuning (IPT)

Recently, IPT was introduced as a combination of ICT and PT to facilitate the knowledge transfer of contextually dissimilar tasks by concatenating soft continuous prompts of the source task with retrieved demonstrations of the target task (39). Singhal et al. (44) introduced the concept of an LLMs' transferability to unseen tasks in classification and NLG medical domains. Nguyen et al. (45) proposed a novel depression screening process based on out-of-domain knowledge transfer methods. Ji (10) introduced an NLP-based suicidal risk detection method based on the sentiment classification capability of LLMs. Gupta et al. (46) explored the LLMs' zero-shot performance on unseen dialogue-related NLG tasks and cross-task generalization in multiple dialogue settings. The same approach was further modified to enhance the cross-task generalization capability of GPT-3 on stress screening (47).

## 4 Applications

Downstream tasks and applications depending on the transferability of soft prompts use in-domain, out-of-distribution, and out-of-domain PE-based mechanisms (48). In-domain prompt transfer adapts an LLM to a specific task within the same domain while out-of-distribution focuses on selecting a different distribution of the same source corpus within the same domain settings (49). Out-of-domain, which is the latest research trend, facilitates transferring LLMs into contextually dissimilar NLP tasks in different domains. In this section, applications of PE in DMH including classification, generation, and question answering tasks are discussed.

### 4.1 Classification task

Anxiety detection, depression detection, and suicidality detection are the most cited application domains of the DMH classification task. Abd-Alrazaq et al. (31) were the first to present a scoping review for n-shot ICL-based prompt engineering techniques in DMH. EMU framework, compatible with passive modalities, was introduced to screen depression and anxiety and the corpus was made publicly available for research purposes (9). Amin et al. (13) analyzed the depression detection capability of ChatGPT using n-shot prompting. Yang et al. (12) explored mental health analysis across five tasks including depression classification and introduced a reliable annotation protocol using emotion-enhanced COT prompting. Mental-LLM was introduced as a SOTA LLM for depression and stress classification using GPT-3.5 and GPT-4 prompting (15). Qi et al. (11) showed suicidality detection in social media posts using zero-shot and few-shot ICL-based prompting. Guo et al. (16) invented a topic modelling framework for depression detection on low-resource data based on handcrafted n-shot prompting. Only a few studies focused on the quality of generated responses by ChatGPT for suicidality detection using n-shot prompting (17). Recently (20), investigated LLM prompting for DMH using large and noisy social media corpora.

### 4.2 Generation task

Considering the reasoning capabilities of LLMs, several generation-based tasks for DMH can be identified. Prompt-based generation is important to predict mental health conditions. Yang et al. (12) showed the sensitivity of LLMs for different input prompts such as *severe* and *very severe* in explainable mental health analysis while mitigating the consequences using few-shot prompting. LLaMA-2 was used as a text augmentation assistant in content generation for mental healthcare treatment planning (50). MentalLLaMA was invented to improve the interpretability of LLMs in DMH (18). Qin et al. (19) introduced a novel COT prompting approach for depression detection and reasoning using zero-shot and few-shot out-of-distribution, which are unseen samples within the same domain, settings. Recently, this was further enhanced using explainable LLM-based techniques to understand psychological state (22). Agrawal (23) improved the explainability and reasoning of the latest generative LLMs in depression analysis using a novel COT prompting framework. Inspired by the Generate, Annotate, and Learn (GAL) framework by (51), a novel suicidality detection framework was introduced to generate synthetic data using LLMs to improve explainability (52). In comparison with classification-based tasks, most of the generation-based tasks use COT prompting as the PE type.

### 4.3 Question answering task

Only a few recent studies have demonstrated question-answering in psychological consultation services and online counselling for mental health professionals. Frameworks such as Psy-LLM, pretrained with LLMs and prompt-tuned with question-answering from psychologists, provide peer support and mental health advice in psychological consultation (53). Liu et al. (54) presented ChatCounselor, an enhanced LLM-based chatbot fine-tuned with domain-specific prompts and demonstrations to reinforce high-quality reasoning and question-answering in DMH. Recently (24), introduced BOLT, an ICL-based framework, to characterize the conversational behavior of clients and therapists.

## 5 Discussion

In this paper, we conducted a short review of how the latest prompt engineering methods in the context of digital mental health are being applied. We discussed three major application tasks to support DMH selecting two major types of PE, n-shot prompting and COT prompting, on ICL, PT, and IPT prompting methods introduced within last five years. In this section, we discuss the challenges, limitations, and future directions in PE for DMH.

There are a few challenges and limitations of PE for DMH. The primary challenge is the scarcity of the data needed to design relevant, accurate, and effective prompts for specific tasks in low-resource and cross-domain settings resulting in low performance during N-shot prompting-based classification and COT prompting-based generation tasks. A few publicly available datasets exist for some PT-based DMH tasks such as bipolar disorder detection, which require specific prompt designs. Even though a few recent studies attempted to mitigate the issue of data scarcity in PT-based tasks using low-resource and cross-domain settings, significant performance is yet to be achieved (55). Designing multiple prompts to improve the performance of N-shot prompting and selecting the most appropriate demonstrations for PT-based and IPT-based knowledge transferring to DMH applications can lead to higher computational requirements resulting scalability issues in LLMs. Although multiple studies recommend soft prompts over handcrafted prompts, it was found that the performance of LLMs tend to overfit due to the nature of bias in soft prompts (56). On the other hand, designing handcrafted prompts requires vast domain knowledge, clinical expertise, and terminology, resulting in uncertainty about better prompt designs for different N-shot prompting-based DMH tasks. In some cases, the performance of LLMs is over-estimated due to in-context information leakage and biased prompts (56). Another challenge is to select the most appropriate demonstrations for cross-model and cross-task transfer using different source and target prompts, to achieve LLM generalization for unseen data in N-shot ICL and COT prompting. Adapting the knowledge of a LLM for depression classification into a different task such as IPT-based depression reasoning is challenging due to the selection of effective demonstrations (57).

Prompt variability and framing plays an important role in maintaining the accuracy and reliability of LLMs in PT-based classification and generation-based tasks (58). An LLMs' probability of generating different predictions for a specific task is high due to the prompt framing effect. A few vulnerability attacks such as prompt leaking and goal hijacking expose confidential details to public scrutiny, by twisting the original task of a prompt, and this must be carefully prevented in DMH COT prompting-based reasoning tasks (59). Preventing adversarial attacks, manipulating LLMs to generate erroneous results using crafted prompts, is also a challenging task even though few attempts have been made to mitigate those using PT-based methods (60). Improving LLMs' interpretability and self-consistency in generation and reasoning tasks in DMH is also identified as a formidable challenge due to its complexity (61).

Using PT-based and ICL-based methods to work with mental health data brings several ethical considerations that need to be carefully addressed. An ethical-legal guidance and clinical validation framework is important to reduce the uncertainty in algorithmic bias, DMH data misuse and to improve LLM transparency and explainability (62). Data anonymization methods and carefully designed prompts should be used to improve the contextual understanding of LLMs mitigating privacy, confidentiality, uncertainty, and accountability issues in ICL-based reasoning. Model reliability should be validated when applying PT-based techniques to improve frozen LLM in-domain knowledge transferability for DMH tasks. Psychological impact and professional autonomy of clinical practitioners, on the other hand, should be carefully considered to assess the quality of prompt designs and in-context examples used for IPT-based out-of-domain DMH tasks.

Prompt automation and intelligence, automating downstream tasks using prompt-driven conversational agents, is a potential direction to enhance the efficiency and accuracy of DMH tasks by processing data more accurately (63). Multimodal COT prompting is an emerging trend to use COT prompting methods for processing multiple forms of mental health data such as text and images to further improve the reasoning capabilities (64). Recently, domain generalization for few-shot settings has been achieved to adapt learned prompts into unseen domains (65). Future research, such as pairing source task prompt embeddings with the in-context demonstrations of another different task and domain shifts with multiple soft prompts, is needed to achieve satisfactory performance in out-of-domain IPT-based task transfer.
